# Animal Models to Test SARS-CoV-2 Vaccines: Which Ones Are in Use and Future Expectations

**DOI:** 10.3390/pathogens12010020

**Published:** 2022-12-23

**Authors:** Gabrielle Gimenes Lima, Amanda Izeli Portilho, Elizabeth De Gaspari

**Affiliations:** 1 Immunology Center, Adolfo Lutz Institute, São Paulo 01246-902, SP, Brazil; 2 Graduate Program Interunits in Biotechnology, University of São Paulo, São Paulo 05508-900, SP, Brazil

**Keywords:** SARS-CoV-2, animal model, vaccine, gold standard, new variants, coronavirus

## Abstract

Since late 2019 and early 2020, with the emergence of the COVID-19 pandemic, scientists are rushing to develop treatment and prevention methods to combat SARS-CoV-2. Among these are vaccines. In view of this, the use of animals as experimental models, both to investigate the immunopathology of the disease and to evaluate the efficacy and safety of vaccines, is mandatory. This work aims to describe, through recent scientific articles found in reliable databases, the animal models used for the in vivo testing of COVID-19 vaccines, demonstrating some possibilities of more advantageous/gold-standard models for SARS-CoV-2 vaccines. The majority of the studies use rodents and primates. Meanwhile, the most adequate model to be used as the gold standard for in vivo tests of COVID-19 vaccines is not yet conclusive. Promising options are being discussed as new tests are being carried out and new SARS-CoV-2 variants are emerging.

## 1. Introduction

The SARS-CoV-2 emerged in late December 2019, and since then, the virus has spread around the world, causing the COVID-19 pandemic [1]. Coronaviruses (CoVs) are single-stranded, spherical viruses that belong to the family *Coronaviridae*, the order *Nidovirales* and the subfamily *Coronavirinae* and are widely distributed in humans and other mammals [2,3]. The subfamily *Coronavirinae* comprises viruses of medical and veterinary importance and contains four genera: alpha-, beta-, gamma-, and delta- coronaviruses (α-, β-, γ- and δ-CoV) [4].

After two years, new SARS-CoV-2 variants have emerged, such as Alpha (B.1.1.7), Beta (B.1.351), Gamma (P.1 or B.1.1.28.1), Delta (B.1.617.2), and the most recent Omicron variant (B.1.1.529) [5]. Given that SARS-CoV-2 is a new virus, therapy strategies were scarce in the beginning of the pandemic. Therefore, the scientific community has studied therapeutic options, with a focus on vaccine preparations, since preventive measures are the key to controlling the disease [6,7].

As it is known, vaccine studies must go through pre-clinical evaluations in animal models. This review aims to describe the main animal models used to develop COVID-19 vaccines so far, their limitations, advantages and how animal studies have contributed to the efforts in controlling SARS-CoV-2.

Vaccines save millions of lives each year, as they help the body develop effective immunity by creating functional and specialized immune cells and antibodies against pathogens. Vaccines are the most effective and economical tools in preventing and controlling infectious diseases [7].

Due to the rapid spread of SARS-CoV-2, the variability among clinical cases, their outcomes, and the high number of deaths, a race to find pharmacological treatments and vaccines was initiated once the World Health Organization (WHO) declared COVID-19 a public health emergency of international concern in January 2020 and a pandemic in March 2020. The structural proteins Spike (S)—which is composed of the subunits S1, where the receptor-binding domain (RBD) lies, and S2—and nucleocapsid (N) have been the main focus as vaccine targets [8,9].

Currently, the WHO COVID-19 landscape has compiled 23 vaccines that are in use around the world. These use different immunization technologies, such as an inactive virus, an adenovirus as a non-replicating viral vector, and mRNA. The vaccine’s efficacy varies from 50.38% to 95% [10].

**Figure 1:**
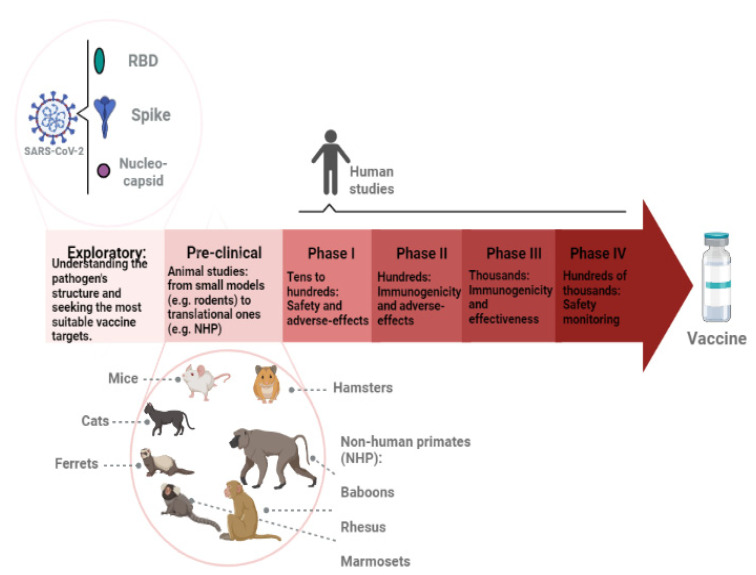
Representation of vaccine development stages. First, exploratory research is conducted to better understand the pathogen. The most conserved and possibly immunogenic antigens are investigated and isolated. New technologies, such as gene sequencing and bioinformatics tools, are applied. In the SARS-CoV-2 case, the main antigens that have been used in vaccine development are the spike, the receptor-binding domain (RBD), and nucleocapsid proteins. The pre-clinical phase involves animal studies. It usually starts in smaller models, such as rodents, then moves onto translational models, such as non-human primates (NHP). The following phases (I, II, III, and IV) focus on human studies. The number of volunteers increases along with the phases. Safety, immunogenicity, and overall effectiveness are analyzed. When thousands of people are being vaccinated, the monitoring continues to verify rare adverse effects.

The licensure of further vaccines would improve vaccine coverage [11]. Studies of new vaccine formulations and platforms are needed, especially in low-income countries, in order to provide access to vaccine manufacture [12]. Figure 1 describes the phases of vaccine development, focusing on the following SARS-CoV-2 aspects: the main targets and the animal models used during pre-clinical stages.

Regulatory agencies require several studies in order to approve a vaccine. Even though there is a current development of in silico and in vitro alternatives, animal studies are still needed to evaluate the mode of action, adverse effects, efficacy, efficiency, and safety, among other parameters [13,14].

The use of animals in scientific research dates back to ancient Greece. However, it was Claude Bernard, around 1865, who launched the principles of using animals as models for study and transposition to human physiology. For this article, “model” is understood as an object of imitation, i.e., something similar. To this end, an animal model should meet the following assumptions: (a) it allows the study of biological or behavioral phenomena of the animal; (b) a spontaneous or induced pathological process can be investigated; (c) the phenomenon, in one or more aspects, is similar to the phenomenon in humans. Among the most commonly used models are rodents (rats, mice, gerbils, hamsters), rabbits, non-human primates (NHP), and some species of fish, amphibians, and invertebrate animals. All research involving this type of experimentation should be subjected to the investigation of an institutional, multidisciplinary, independent, and autonomous ethics committee, in order to avoid the misuse and mistreatment of laboratory animals [13,15].

In this context, when it comes to vaccine development for COVID-19, the use and choice of reliable and well-characterized experimental models are important for the rapid advancement of research and, consequently, the registration and availability of the vaccines. Although several animal models have been developed for human coronavirus infection, including SARS-CoV, MERS-CoV, and now SARS-CoV-2, to date, none of these fully represents the pathology or clinical symptoms of human infection [16]. As stated, there is no questioning the importance of using animal models, ethically, to understand the pathophysiology of SARS-CoV-2, and so appropriate animal models that mimic the biology of human SARS-CoV-2 infection are urgently needed. However, one should always keep in mind that the emergence of new variants can lead to a delay in testing in animal models, since the selected viruses must first be isolated and characterized in vitro and then followed by animal model analysis. Nevertheless, in vitro studies cannot completely simulate human pathophysiology, as immune components are very complex, but despite the differences between animal and human models, critical related information can still be discovered. The limitation of using a small animal model is the intrinsic biological differences between humans and rodents, and with this, the viruses must be adapted to the animals, or the animals must be genetically manipulated to recapitulate the human system, and there is also the difference in life span for monitoring the disease. In large animal models, replicating the pathogenesis of human diseases is easier, because they are physiologically, immunologically, and genetically more closely related, but there are high costs and resources involved, which limit the number of animals that can be included in a study, and there is wide variability in genetic backgrounds [17,18,19,20,21].

Since each animal model has its strengths and limitations, we recommend selecting the optimal animal model in relation to the research questions at hand [22].

## 2. Materials and Methods

This article is a narrative review. The scientific articles cited were found through the platforms PubMed and Web of Science. The keywords “SARS-CoV-2”, “Animal Model”, “Vaccine”, “Gold Standard”, and “New variants” were used, and manuscripts were selected considering the relevant aspects for this revision. For the PubMed platform, the following filters were used: “Publication date”, selecting articles from 2020 and 2022; “Text Availability”, opting for articles available as “Free Full Text” and “Full text”; “Article Attribute” with “associated data”; with “Species”, the option “other animals” was chosen, since we are dealing with non-human animal models; and for “Language”, “English” and “Spanish” were selected. For the Web of Science platform, the filters “Open Access” and “Year of Publication” were used, selecting only articles from 2020–2022. With exclusion criteria of the articles found, 110 articles were selected. This literature review research was expected to last 5 months. In the first week, the survey of the theoretical framework was carried out; in the second week, the literature review was performed; in the third week, the preparation of the pre-textual and post-textual elements that compose the work was conducted; in the fourth week, the revision was completed; and in the fifth week, the final correction of the document was achieved.

The following sections will discuss animal models that have been used in SARS-CoV-2 research and the results of related studies.

## 3. Results

### 3.1. Laboratory Animals

#### 3.1.1. Mice

One of the major challenges of these animals as experimental models is their resistance to SARS-CoV-2. To overcome this limitation, an adapted virus can be used to infect these animals. Another approach is the use of transgenic animals developed to study SARS-CoV-2. However, since much research is being conducted on COVID-19, the high demand can make this alternative unfeasible [16,23]. To address this deficiency, Leist, Schäfer, and Martinez [24] reviewed in their research a way to sensitize mice by delivering human ACE2 (hACE2) by adenovirus and adeno-associated virus (AAV) transduction to immunocompetent and immunodeficient animals. Although AAV delivery of hACE2 is not specific for cells that are permissive to SARS-CoV-2 in humans, this model may be suitable for testing antibody and drug therapies [24]. Gene-editing technology by CRISPR / Cas9 was also applied in the development of a hACE2 knock-in humanized mouse that developed interstitial pneumonia [25] and intragastric inoculation [26].

Sun et al. [23] developed a transgenic mouse model by delivering hACE2 exogenously with a replication-deficient adenovirus (Ad5-hACE2). It was observed that these animals infected with SARS-CoV-2 showed raised fur and difficulty breathing 2 days after infection. In addition, there was a loss of about 20% in body weight, with the virus severely affecting the lung tissue 4-6 days following infection. Although these symptoms gradually decreased, a variety of lesions in the lung tissue were noted by microscopic analysis. In the same study, the use of treatments for SARS-CoV-2, such as immune plasma therapy, avoided weight loss, histological changes in lung tissue, and accelerated viral clearance. Although the sensitized mice did not develop severe disease or extrapulmonary manifestations, which are important aspects of the disease [23,25].

Exogenous delivery of hACE2 resulted in viral replication in the mouse lung with clinical symptoms. However, it can trigger individual variations in hACE2 expression and cellular distribution [25]. 

As reported by Sun and colleagues, SARS-CoV-2 could replicate in the upper and lower airways of BALB/c transgenic mice expressing hACE2, regardless of the animals’ age. Meanwhile, elderly mice presented a more severe infection, as observed in humans [27]. Another study examined infectivity and pathological changes in hACE2 transgenic mice infected with SARS-CoV-2, demonstrating several symptoms that also recall human infection. Thus, surviving mice were protected from reinfection. An advantage of rodent models is their abundant availability and low cost, which allows more animals to be used per study, increasing the statistical power [26]. 

In addition, vaccines using various platforms (mRNA, viral vectors, recombinant subunits, and inactivated virus) [28] have been tested in these animals inducing protective immune responses [25]. The interesting platform of Counoupas et al. [29] verified in this model that a Spike protein vaccine adjuvanted by the bacille Calmette-Guérin (BCG) induced high neutralizing antibody titers, Th1 cytokines, and T follicular helper cells, and thus protection of the lungs. The modified vaccinia virus Ankara was explored as a delivery system for the Spike protein as well, inducing high antibody titers and protecting hACE transgenic mice from viral challenge [30]. Immunization using the parainfluenza virus 5 expressing the Spike antigen protected the same mouse model from viral challenge following a single intranasal dose [31]. 

Due to amino acid differences between murine and hACE2, inbred mouse strains do not support high titer viral replication of the SARS-CoV-2 virus. Therefore, several transgenic and knock-in mouse models, as well as viral vector-mediated hACE2 delivery systems, have been developed. In this work, it was shown that K18-hACE2 mice replicate the virus at high titers in both the lung and brain, leading to lethality. The K18-hACE2 model provides a rigorous option for testing the ability of vaccines and antivirals to protect against disease [21].

However, animal models of K18, CAG, HFH4, and knock-in mice show neuroinvasion and high titer replication in the brain, which probably leads to lethality [32].

Therefore, the use of mice is suitable for the evaluation of vaccine performance against viral replication in the lungs and pathogenesis, facilitating the in vivo screening of therapeutics and vaccine development [24].

#### 3.1.2. Syrian Hamster *(Mesocricetus Auratus)*

Golden Syrian is a suitable model for reproducing human pathogenesis and prophylaxis against SARS-CoV-2 infection [24,25]. Replication occurs at high titers in the respiratory tract accompanied by pulmonary pathology, pulmonary edema, and interstitial pneumonia, because of increased viral load and extensive apoptosis [25,26]. However, it may result in the transmission of the virus to other animals via aerosols [25,26,33]. The transmissibility of SARS-CoV-2 can be analyzed by generating neutralizing antibody responses that protect against pulmonary viremia, as well as the passive transfer of convalescent serum to naïve animals [24,25].

Rosenke et al. [34] studied hamsters inoculated intranasally and verified that the SARS-CoV-2 S protein effectively binds to the Syrian hamster ACE2 receptor; that these animals are susceptible to SARS-CoV-2, presenting broncho-interstitial pneumonia and high viremia in the lungs; that neither the age nor the sex of the individuals affected the severity or outcome of the disease; and that those hamsters lacking interleukin (IL)-2 receptor gamma subunit showed persistent infection. The infected animals showed consecutive weight loss, raised fur, and breathing issues. All animals presented lung lesions, and viral RNA was detected in all tissues examined. The article also highlights the lack of systemic response and the absence of the respiratory decompensation associated with acute respiratory syndrome [34]. In contrast, a section of the literature shows sex differences in lung imaging and SARS-CoV-2 antibody responses in a COVID-19 Golden Syrian hamster model [34].

In other studies, hamsters inoculated with SARS-CoV-2 showed weight loss, lethargy, raised fur, arched back posture, rapid breathing, changes in inflammation, cellular expression of N protein expression, and elevated viral load [25,26,32]. qPCR of lung samples demonstrates an early IFN response and an elevation of IL-6. SARS-CoV-2 can replicate in the brain or olfactory bulb of Syrian hamsters and damage olfactory sensory neurons [25,32].

Administration of neutralizing anti-RBD antibodies effectively reduced viral load in the Syrian hamster model [25].

A vector-based vaccine of adenovirus expressing the S protein of SARS-CoV-2 induced neutralizing antibodies and protected hamsters against severe disease. Nevertheless, there are other vaccines that have used this model [35]. Similar results were obtained when a live-attenuated [36] and a nanoparticle [37] nasal COVID-19 vaccine were tested in this animal model. The ChAdOx1-nCov vaccine, developed by AstraZeneca/Oxford, was also tested in hamsters, protecting the animals from live virus challenge [38]. Therefore, hamsters can be used as a model for studying therapeutics, immunotherapy, and immunoprophylaxis [26]. Thus, it is relevant to study the natural infection of these animals, which could even be considered a predetermined experimental model. 

### 3.2. Non-Human Primates

Considering the homology with the host receptor ACE2, non-human primates would score a higher level of susceptibility to SARS-CoV-2 infection than rodents, which would make them a more suitable model to translate experimental data [39].

NHPs are phylogenetically related to humans and share a wide range of viral pathogens, often mimicking the clinical presentation of human infections, and the possibility to perform studies under standardized conditions is a positive aspect to performing vaccine protection tests. In addition, their immune system and respiratory system anatomy are very similar to humans. To date, Rhesus and Cynomolgus are the best-characterized species for COVID-19 drug and vaccine research; an important caveat is that SARS-CoV-2 infection in Cynomolgus resembles only mild-to-moderate cases in humans. As the development of COVID-19 vaccines has advanced at a rapid pace, there is also a current shortage of NHPs [39]. However, in NHP models, it has been reported that after a primary exposure, Rhesus macaques were protected against SARS-CoV-2 reinfection [40].

The NHP model has been used to demonstrate immunogenicity and protective efficacy against SARS-CoV-2 with several vaccine candidates, because the high level of protection achieved with vaccines is clinically relevant and similar to the results of human trials [41].

Further studies are needed to investigate the impact of emerging SARS-CoV-2 variants on vaccine candidates tested in NHPs. When NHP models are compared, Rhesus monkeys (*Macaca mulatta*) showed bodyweight loss, severe pneumonia, significant symptoms, high virus shedding, multiple organ infection, the higher susceptibility to SARS-CoV-2 and disease severity. African green monkeys (*Chlorocebus aethiops*) showed severe pneumonia, significant symptoms, high virus shedding, multiple organ infection, and high susceptibility to SARS-CoV-2 and disease severity, following Rhesus. Cynomolgus monkeys (*Macaca fascicularis*) showed mild symptoms, moderate virus shedding, body weight loss, mild-to-severe pneumonia, moderate susceptibility to SARS-CoV-2 and disease severity. Common marmosets showed no symptoms, no pneumonia, low virus shedding, and overall lower susceptibility to SARS-CoV-2 and disease severity [42,43,44].

#### 3.2.1. Marmosets

Species of marmosets have been used to study infections with SARS-CoV and MERS-CoV viruses. However, despite the detection of viral RNA up to day 14 in blood, nasal, tracheal, and rectal swabs, marmosets showed some resistance to infection with SARS-CoV-2, and no seroconversion, pneumonia, or traces of the virus in other tissues were observed [45]. However, some studies point out that even showing resistance, these animals have symptoms such as diarrhea, pneumonitis, fever, watery stools, and hepatitis [26,46]. Even though there are fewer studies using this model when compared with other NHPs, an inactivated vaccine induced neutralizing antibodies that persisted for 50 weeks in marmosets [47].

#### 3.2.2. Cynomolgus *(Macaca fascicularis)*

The Cynomolgus *(Macaca fascicularis)* demonstrated pathological changes in the lung, such as diffuse alveolar damage that coincides with co-localization of SARS-CoV-2 antigen expression, punctate pulmonary hemorrhage, and lung lesions [42]. Cynomolgus macaques have effective virus transmission to other animals and can be used as a model of transmission. The Novavax vaccine, composed of the Spike protein adjuvanted by Matrix-M™ (a saponin-based adjuvant), was tested in this model, presenting neutralizing antibodies; thus, the animals were protected from respiratory infection and pulmonary disease upon intranasal and intratracheal challenge [48]. The ferritin-Spike nanoparticle proposed by the Army Institutes of Research from the United States also used the Cynomolgus model, revealing robust humoral and cellular immune responses, along with reduced lung lesions after SARS-CoV-2 infection [49].

#### 3.2.3. African Green Monkey *(Chlorocebus sabaeus)*

African green monkeys *(Chlorocebus sabaeus)* had lung lesions, similar to humans, after an infection. Respiratory epithelial cells and type II pneumocytes are the target cells of SARS-CoV-2. There was detectable viral RNA and infectious virus in nasal secretions and saliva in these monkeys [24,25]. Although we could not find examples of vaccine testing in this species, the model was used to study the intra-host evolution of SARS-CoV-2. Such knowledge could be used to explore the more likely mutations in controlled environments and test vaccine efficacy in preventing persistent shedding of SARS-CoV-2, which has been observed in some patients [50,51]. Moreover, viral shedding in the guts was also investigated in African green monkeys, supporting the hypothesis that inducing mucosal immunity would be beneficial for future COVID-19 vaccines [50,52,53].

#### 3.2.4. Rhesus Macaques *(Macaca mulatta)*

Along with Cynomolgus, this specie were the first models used to study SARS and MERS. To date, these animals are the most informative due to the similarity between their anatomy and that of humans. Studies have pointed to the existence of neutralizing antibodies specific for SARS-CoV-2 after primary infection that may protect against reinfection. The Rhesus monkey mimics human infection well because of the similarity between the monkey ACE2 to hACE: the receptors share 23 amino acid residues in the protein region that makes close contact with the RBD of SARS-CoV-2 [24,25].

Since age is one of the risk factors for severe COVID-19, the response of aged rhesus monkeys to intratracheal inoculation with SARS-CoV-2 was comparable to younger controls, and there were indications of mild interstitial infiltrates in younger animals and alveolar flooding in aged monkeys. Studies point out that the association of virus infection with age and its replication mainly occur in the lungs and nasopharyngeal swabs of the elderly more than in young rhesus monkeys [19]. Thus, a valuable feature of NHP models is their potential use for modeling age-dependent and disease progression that represents severe cases of COVID-19 [25,33].

Different vaccine platforms were tested in Rhesus macaques. An inactivated vaccine resulted in increasing IgG and neutralizing antibodies titers, and thus viral clearance following intranasal challenge [54]. A vaccine of adenovirus expressing the spike protein induced, after a single dose, neutralizing antibodies that supported viral clearance in bronchoalveolar lavage and nasal swabs after SARS-CoV-2 intranasal and intratracheal challenge [55]. A DNA-vaccine encoding the Spike provided not only humoral, but also cellular immune response and prevented the macaques from infection [56]. 

Moreover, studying the vaccine and the natural-induced immune response in Rhesus macaques provided data pointing to the protective role of CD8+ cells, supporting the idea that vaccine development should aim not only for humoral, but also cellular immunity [57].

#### 3.2.5. Baboons

Tian et al. [23] developed a SARS-CoV-2 subunit vaccine from the highly immunogenic S protein tested in mice and baboons. After determining that low doses of the vaccine induced protective neutralizing antibodies and multifunctional antigen-specific T cells in mice, the immunogenicity of the vaccine in adult baboons was compared with those of human patients who had recovered from COVID-19. It was observed the vaccinated baboons developed comparable or even higher levels of functional antibodies than COVID-19 patients [23].

### 3.3. Wildlife

#### 3.3.1. Ferrets

In early 2020, it was discovered that ferrets and humans share critical amino-acid residues in their ACE2 sequences. Other common aspects regarding COVID-19 are viral elimination and replication in saliva, nasal tissues, lung, and intestinal tissues, leading to the detection of viral RNA in fecal debris. Therefore, ferrets may be a suitable model for the dissemination of asymptomatic or mildly symptomatic SARS-CoV-2 in the human population [25,58,59]. 

Ferrets are commonly used in immunopathological studies to evaluate treatments and vaccines [59]. Virus replication was reported in the lungs and histological changes such as pneumonitis following intranasal inoculation [26]. The lungs of ferrets infected with SARS-CoV-2 exhibited severe pulmonary lymphoplasmacytic perivasculitis and vasculitis 13 days after infection [59]. Ferrets infected with SARS-CoV-2 showed mild clinical signs, such as elevated body temperature, and the virus presented upper respiratory tract (UTR) tropism [25,32]. There have also been studies confirming similar inflammatory cytokine profiles following SARS-CoV-2 infection of human and ferret airway cells, including expression of genes encoding IL-6, IL-1β, and the chemokine ligands CCL2 and CCL8 [24,60].

There have been reports of SARS-CoV-2 transmission from infected to naïve ferrets in co-housed animals and via indirect contact, suggesting transmission by saliva droplets or potentially airborne transmission [24,25,26]. Therefore, these animals can be used to understand transmission dynamics and specific mutations. In fact, the study of An and colleagues [31] verified that an intranasal parainfluenza-vectored COVID-19 vaccine prevented viral infection and transmission between ferrets. The susceptibility of ferrets to SARS-CoV-2 has led to the use of this model in evaluating the efficacy of antiviral therapy and vaccination [25,26].

#### 3.3.2. Pangolin

Animals that tolerate infections are capable of carrying a high load of infectious agents and may thus be relevant intermediaries of transmission to other species. For example, bats contributed to the spread of Severe Acute Respiratory Syndrome (SARS) and Middle East Respiratory Syndrome (MERS) [61].

In 2020, it was suggested that the Malay Pangolin (*Manis javanica*) would have been a SARS-CoV-2 intermediary host [61,62,63,64,65]. Therefore, understanding how pangolins can survive and tolerate coronavirus may result in new treatment options for humans [61]. 

ACE2 is conserved in pangolins, and the coronaviruses isolated in these animals presents RBD in their Spike protein. However, these animals present several differences in their immune system, such as deficiencies in genes encoding the Toll-like receptor (TLR)-5 (a bacterial flagellum receptor); the IFNE (Interferon epsilon, an antiviral in epithelial cells); the IFIH1 (interferon-induced with helicase C domain 1, a cytoplasmic RNA sensor that activates an innate immune response to coronavirus infection) [61]; and Retinoic acid-inducible (RIG)-gene I, another sensor against viruses [61]. The scientific community could explore whether these genetic differences in pangolins are protective against COVID-19; as such, further investigation would be relevant to understanding how the defense against coronavirus works in the pangolin model and whether these findings could be employed to design vaccines that modulate similar mechanisms of protection. In addition, therapeutic strategies could be investigated as well. For example, it was hypothesized that the loss of IFIH1 in pangolins may have provided an evolutionary advantage by reducing inflammation-induced damage to host tissues and contributing to the tolerance of viral infections in pangolins [61]. As it was further proved, reducing inflammation is an important measure to reducing COVID-19 burden for severe patients [66].

### 3.4. Felines 

#### Cats

Animal studies have also confirmed the susceptibility of domestic cats to infection, as well as tigers in zoos in the United States [26]. Specific antibodies against SARS-CoV-2 have been found and ACE2 in this case also contributes to infection [27,59]. Therefore, feline studies of SARS-CoV-2 deserve attention [24,25,26,27,59]. This animal model is suitable for vaccine or antiviral efficacy studies against SARS-CoV-2, since, in cats, the virus replicates in the nose and throat, causing deeper inflammatory pathology in the respiratory tract, and airborne transmission occurs between pairs of cats [59]. Thus, implementing new studies to find out whether the severe disease can be replicated in cats should be useful for testing vaccine efficacy and veterinary drug development [24].

Finally, a compilation of characteristics of the animal models most often used to study SARS-CoV-2 is presented in Table 1.

## 4. Discussion

### 4.1. Vaccines Used Worldwide and Animal Models Tested

Animal models are needed to evaluate the safety and efficacy of new vaccines and therapeutics. Studies with the Pfizer vaccine (pre-fusion SARS-CoV-2 Spike RNA) in BALB/c mice and rhesus macaques reported that the vaccine was highly immunogenic and prevented lung infection of animals following virus challenge [89].

Sinovac Biotech Ltd. developed an inactivated SARS-CoV-2 vaccine called CoronaVac. In pre-clinical trials, different animal models were used: BALB/c mice and Wistar rats provided the primary information on safety and immunogenicity, and further evaluation in rhesus monkeys confirmed the data [104]. NVX-CoV, from Novavax, is an S-Trimer-based nanoparticle vaccine that was tested in BALB/c mice transduced with hACE2 and in olive baboons to supplement immunogenicity data [104]. ChAdOx1-S, developed by AstraZeneca, was tested in inbred (BALB/c) and outbred (CD1) mice and in White–Landrace–Hampshire cross-bred pigs [104]. Inovio Pharmaceuticals initiated pre-clinical and clinical trials of a DNA vaccine (INO-4800) against COVID-19 and a two-dose scheme of it in Rhesus monkeys [104].

The World Health Organization (WHO) currently indicates four species as reproducible COVID-19 models: NHPs, ferrets, hamsters, and mice, demonstrating the importance of using the animal model even today [105].

### 4.2. Gold Standard

NHP models of viral diseases are considered the gold standard for modeling human pathogenesis and for testing clinical interventions due to their anatomical/physiological and genetic/phylogenetic similarity to humans [24,25]. These models recapitulate the symptoms of COVID-19 and can be used later to elucidate therapy for SARS-CoV-2 infection [26].

Some studies suggest that species such as Old-World monkeys would be highly susceptible to infection, whereas most rodents are less likely to be infected [27].

However, the results obtained in species with a small number of animals may not be consistent due to biological variability and other parameters, such as availability, affordability, and suitability of the models within the scientific community, which should be considered [26]. For rodent models, though rats and mice need to be adapted for use as an experimental model for SARS-CoV-2 [21], hamsters are highly susceptible to infection, usually manifest variable symptoms, readily transmit the virus to other animals, and develop a rapid virus-specific immune response, as seen in cats and ferrets [59].

It should also be noted that genetically inbred animal models are of great value for qualifying antibodies and vaccines to learn how genetic variation in the host affects the performance of treatments, and whether the age and sex of the animals influence the outcome. Outbred mice, on the other hand, reflect the genetic diversity present in the human population [24].

Wild-type mice require modification of the SARS-CoV-2 RBD to mediate productive infection and pathogenesis [24]. Those infected with SARS-CoV mostly do not exhibit severe clinical signs due to the discrepancy between mouse and human ACE2 (human angiotensin I-converting enzyme 2) (hACE2) [21,26], except for the elderly or immunocompromised; moreover, disease severity varies according to tissue distribution and the level of hACE2 transgene expression. Thus, transgenic mice expressing human ACE2 have been developed to be animal models for the development of SARS-CoV vaccines [21,25]. Antiviral research has also been used to evaluate the prophylactic potential of monoclonal antibodies against SARS-CoV-2 infection, as well as the efficacy of the SARS-CoV-2 vaccines [24,25].

### 4.3. Second-Generation Vaccines: Author’s Point of View

During the pandemic, the importance of devising containment strategies and vaccine development came into focus. However, the post-pandemic scenario is also of paramount importance to contain new outbreaks and possible new pandemics that may arise in the future. There are still data limitations regarding COVID-19, and it is still uncertain how long the vaccines remain with active immunologic memory. Therefore, it is of great value to analyze the need for a new generation of immunizing preparations and booster doses to induce a robust response against SARS-CoV-2 variants.

The control of the SARS-CoV-2 pandemic is in a public health alert mode because of the emergence of variants of concern with higher transmission efficiency and/or virulence.

However, there are studies demonstrating that some neutralization tests already work for these new variants to date (2C08-like antibodies can be induced by SARS-CoV-2 vaccines and mitigate resistance by circulating variants of concern [106,107,108] in chimpanzee and murine models; intranasal immunization provides protection against the ancestral SARS-CoV-2 and two VOC, B.1.1.7 and B.1.351.). Thus, it has been described that hamsters and mice can be affected by new variants [77]. Recently, monoclonal antibodies identified from human memory B cells were tested in a BALB/c model and protected the mice from Beta variant [109].

This study summarizes the findings related to virulence, transmission, and cross-protection in animal models of mice, non-human primates, and ferrets, but more studies are also needed, because there may be an emergence of new variants that will lead to a delay in testing in animal models [110]. It is also worth noting that it is necessary to understand the patterns of immune response after infection with the variants of concern, and further studies are needed.

The second-generation vaccines are important in providing long-term protection with a single immunization. This characteristic could be improved by inducing a T-lymphocyte activation, which would result in adequate differentiation of: (i) CD8+ effector cells; (ii) CD4+ helper cells to orchestrate the cytokine environment and support B cell maturation; (iii) and, finally, memory cells. Considering this, the immune response would be favored by different mechanisms of defense and a longer immunologic memory. It is expected that animal models will support such studies. Thus, another improvement would rely on formulating vaccines that do not require preservation at very low temperatures, as these vaccines would facilitate large vaccination campaigns in geographic regions with limited healthcare infrastructure and resources.

## 5. Conclusions

The urgent need for COVID-19 vaccines has led to unprecedented collaboration between developers, manufacturers, and distributors, in conjunction with governments and academics. As a fundamental part of scientific research, animal models aid in understanding the pathophysiology of diseases and in evaluating preventative methods and treatments, such as vaccines, and they are essential in disease management, even more so when dealing with a pandemic. The predictive value of these animal models depends on their ability to reproduce the characteristics of human disease.

Using various model systems, such as cellular and animal models, along with clinical data from patients, will be of great importance for vaccine development, and it is important to note that the severity of the disease may vary according to the route of inoculation, the dose of inoculation, the age of the animal used, as well as the SARS-CoV-2 isolate, and so an ideal animal model should be standardized in order to analyze clinical symptoms and pathogenesis for the development of therapeutic strategies.

## Figures and Tables

**Table 1:** Characteristics of the most commonly used animal models for SARS-CoV-2.

Animal Model	Advantages	Disadvantages	Susceptibility to Infection	Severity of Infection	Symptoms	Pulmonary Pathology ^a^
Mice [23,27,67,68,69,70,71,72,73,74,75,76,77,78,79,80]	Small size, fast reproduction, wide range of research tools available, well-characterized immune responses, increased throughput	Requires transgenic expression of hACE2 or viral adaptation for infection, differences in lung physiology and immunology compared to humans	Adaptation for use in the experimental model is required	Mild-to-lethal	Difficulty breathing, weight loss, asthenia	Yes
Hamster [77,81,82,83,84,85,86,87,88]	High homology with hACE2, demonstrated substantial inflammation and lung injury	Not widely used, limited research tools available	High (elderly are more susceptible)	Mild-to-moderate	Tachypnoe, weight loss, asthenia, frowning skin, intestinal inflammation	Yes
Non-human primates [89,90,91,92,93,94,95,96,97]	Phylogenetically close to humans, extensively used in viral infections research	Low performance, presents advanced cognition, additional ethical concerns	High (elderly are more susceptible)	Mild-to-moderate	Fever, decreased appetite, dehydration, irregular respiration, Tachypnea, dyspnea, asthenia	Yes
Ferrets [98,99,100,101]	Allows for the study of mild symptoms (e.g., cough), previously used in viral infection research	Unclear whether it can mimetize SARS-CoV-2 severe lung infection and edema	High	Asymptomatic-to-mild	Fever, rhinorrhea, cough, sneezing, decreased appetite, weight loss, asthenia	Yes
Cats [59,102,103]	Lethality with pulmonary edema and natural infections reported	Not widely used in pathology studies, aggression and unpopularity in use as laboratory animal	High	Mild-to-lethal	Fever, apathy, respiratory, digestive and neurologic signs	Yes

^a^ Pulmonary pathology refers to pneumonia and inflammatory infiltrates.

## Data Availability

This manuscript is a narrative review, and no original data were created. The articles used to write this revision are referred to in the bibliography.
